# Copper Isotope Ratio Measurements of Cu-Dominated Minerals Without Column Chromatography Using MC-ICP-MS

**DOI:** 10.3389/fchem.2020.00609

**Published:** 2020-07-16

**Authors:** Yan Zhang, Zhian Bao, Nan Lv, Kaiyun Chen, Chunlei Zong, Honglin Yuan

**Affiliations:** State Key Laboratory of Continental Dynamics, Department of Geology, Northwest University, Xi'an, China

**Keywords:** Cu isotope, Cu-dominated minerals, column chromatography, MC-ICP-MS, C-SSBIN

## Abstract

This study performed a series of comparable experiments (with or without column chromatography) to evaluate whether non-deviated Cu isotope ratios can be obtained directly by Nu Plasma II multi-collector inductively coupled plasma-mass spectrometry (MC-ICP-MS) using standard-sample bracketing with Ga as internal mass bias correction model (C-SSBIN) without column chromatography. Twelve Cu-dominated minerals (copper plate, native copper, chalcopyrite, bornite, chalcocite, digenite, covellite, tetrahedrite, azurite, malachite, atacamite, and cyanotrichite) displayed little drift in δ^65^Cu values compared with those of minerals with column chromatography, with Δδ^65^Cu_without−with_ ranging from −0.04 to +0.02‰. This means that Cu isotope ratios in Cu-dominated minerals can be achieved without column chromatography, due to the simple matrix and the stability of the machine by using C-SSBIN mode. The acidity and internal standard concentration mismatch effects, as well as the matrix effect, were strictly assessed by Nu Plasma II MC-ICP-MS in a wet-plasma mode in the State Key Laboratory of Continental Dynamics (SKLCD). Finally, a long-term reproducibility of better than ±0.03‰ [*n* = 38, 2 standard deviations (2s)] were achieved by repeatedly measuring chalcopyrite without column chromatography over 4 months.

## Introduction

Copper (Cu) is a transition metal element and has two stable isotopes, 63Cu and 65Cu with relative abundances of 69.17 and 30.83% in nature, respectively (Walker et al., 1958; Shields et al., 1965). Cu isotopic fractionation occurs in many aspects of geoscience processes, making Cu isotopic ratios an effective tracer in the fields of mineral deposits (Zhu et al., 2000; Larson et al., 2003; Zheng et al., 2019), biogeochemistry (Zhu et al., 2002; Navarrete et al., 2011; Weinstein et al., 2011), soil-forming process in supergene environment (Fernandez and Borrok, 2009; Bigalke et al., 2011), paleoceanography (Fru et al., 2016; Liu et al., 2019), and moon evolution (Williams and Archer, 2011; Xia et al., 2019). Lots of research have shown Cu isotope ratios are greatly heterogeneous in Cu-bearing ore minerals with δ^65^Cu ranging from −16.5 to +9.98‰ (Maréchal et al., 1999; Larson et al., 2003; Mason et al., 2005; Mathur et al., 2005, 2009a, 2010; Markl et al., 2006; Asael et al., 2007). Therefore, the Cu isotope ratio is useful to trace the ore-forming source, determine metallogenic temperature, and study metallogenic system evolution.

To precisely and accurately determine the Cu isotope ratios, there are two steps for natural samples: column chromatography and mass spectrometry. Because instrumental mass fractionation could be caused by matrix elements in the analytic process, column chromatography is a prerequisite for mass spectrometry. Moreover, column chromatography can reduce or eliminate possible polyatomic interference and doubly charged interference, such as ^47^Ti^16^O^+^, ^27^Al^36^Ar^+^, ^23^Na^40^Ar^+^, ^45^Sc^18^O^+^, ^126^Te^++^, ^126^Xe^++^ on ^63^Cu, and ^49^Ti^16^O^+^, ^25^Mg^40^Ar^+^, ^130^Xe^++^, ^130^Te^++^, ^130^Ba^++^ on ^65^Cu. Previous studies have developed column chromatography with diverse cation-exchange resins, acids, and columns with a high Copper yield (>99%) (Maréchal et al., 1999; Maréchal and Albarède, 2002; Borrok et al., 2007; Liu et al., 2014; Zhu et al., 2015; Hou et al., 2016). Maréchal and Albarède (2002) indicated that the Cu isotope fractionation happened during column chromatography. Therefore, to reduce the fractionation of column chromatography and obtain accurate Cu isotope ratios, a complete recovery must be realized. Recently, some studies reported separation of Cu from igneous rock samples by using one-step anion-exchange method with strong anion resin AG-MP-1M (Liu et al., 2014; Hou et al., 2016). Liu et al. (2014) used 8 N HCl added 0.001% H_2_O_2_ to separate Cu from the igneous rock and avoid the fractionation of Cu isotope during column separation. They found the Cu isotope composition in the igneous rock is heterogeneous and could be used to trace the process of high-temperature magma evolution. Hou et al. (2016) used 8.5 N HCl mixed with 0.03% H_2_O_2_ to purify Cu in the igneous rock and separated Co and Cu effectively, with the Co/Cu in purified solution was <0.02.

Besides column chromatography, mass spectrometry also plays a significant role in Cu isotope analysis. Walker et al. (1958) and Shields et al. (1965) measured the Cu isotope ratios in natural samples using thermal ionization mass spectrometry (TIMS) firstly. However, because of the limitation of analysis precision (1–1.5‰), no variation in ^65^Cu/^63^Cu ratios of geological samples was found. In recent years, the Cu isotopic ratios can be measured by MC-ICP-MS with higher analytical accuracy and precision compared with TIMS (Maréchal et al., 1999; Zhu et al., 2000, 2002; Archer and Vance, 2004). However, MC-ICP-MS suffers from much larger mass bias/isotope fractionation (mass-dependent and mass-independent fractionation) than TIMS in the measurement of isotopic ratios, and thus selection of the suitable mass bias correction method is very important for different isotopes (Yang, 2009; Yang et al., 2018). For copper isotope ratios determination, the most commonly used models to improve the analysis accuracy and precision include the straightforward standard-sample bracketing (SSB), combined standard-sample bracketing with internal standard (C-SSBIN) and regression mass bias correction (Maréchal et al., 1999; Zhu et al., 2000, 2002; Archer and Vance, 2004; Yang, 2009; Larner et al., 2011; Hou et al., 2016; Yang et al., 2018). For example, the pioneering work of Maréchal et al. (1999) displayed that instrumental mass bias could be corrected by combining Zn-doped with SSB method, so it was possible to obtain precision of better than 0.1‰ for Cu isotopic ratio using MC-ICP-MS. Zhu et al. (2000, 2002) and Archer and Vance (2004) also determined the Cu isotopic ratios using this approach. Larner et al. (2011) used Ni as an internal standard element to correct instrumental mass bias during Cu isotope analysis and obtained accurate Cu isotope ratios with reproducibility of ±0.04‰ for the pure standard solution and ±0.15‰ for biological samples. Recently, Hou et al. (2016) used Ga as an internal standard with the C-SSBIN mass bias correction model to correct short-term fluctuations and effectively improved the precision approximately five-folds from 0.05 to 0.01‰.

Several recent studies have attempted to determine Cu isotopic ratios by using Laser ablation (LA) combined with MC-ICP-MS, indicating the potential of analyzing Cu isotopic compositions without chemical chromatography (Jackson and Günther, 2003; Graham et al., 2004; Ikehata et al., 2008; Lazarov and Horn, 2015). For example, Jackson and Günther (2003) firstly applied nanosecond (4 ns, 266 nm Nd: YAG; 12 ns, 193 nm ArF Excimer) LA-MC-ICP-MS to study Cu isotope of samples, and observed large mass discrimination effect relative to the Cu isotopic ratios measured by conventional solution nebulization MC-ICP-MS. This effect was related to particle size-related fractionation and caused by the incomplete violatilization of all particles by ICP. A near-infrared femtosecond (780 nm, 227 fs) LA-MC-ICP-MS was used to determine the Cu isotopic ratios of metals and Cu-dominated minerals with a precision of better than 0.14‰ (2SD). Compared to the Cu isotopic ratios measured by solution nebulization MC-ICP-MS, reliable Cu isotopic results can only be determined by using matrix-matched calibrated standards (Ikehata et al., 2008). With the development of femtosecond laser ablation system, deep ultraviolet-femtosecond (194 nm, 200 fs) LA-MC-ICP-MS was used to determine the Cu isotope ratios in native copper, Cu-bearing sulfides, oxides, and carbonates by straightforward standard-sample bracketing with NIST SRM 976 (Lazarov and Horn, 2015). Appropriate low fluence (~0.3 J cm^−2^) was used to measure Cu isotopic ratios in samples *in situ* with an analytical precision of better than 0.1‰ combining using Ni as an internal standard with a non-matrix-matched standard. In contrast, Ikehata and Hirata (2013) reported poor accuracy of the results obtained by ultraviolet-femtosecond (260 nm, 227 fs) LA-MC-ICP-MS with non-matrix-matched calibration. Therefore, careful selections of standards are necessary for accurate *in situ* Cu isotope ratios analysis of Cu-bearing minerals. The above studies suggested measuring Cu isotope ratios without column chromatography is possible, depending on strict control of fsLA-MC-ICP-MS parameters, such as ablation approach, pulse width, energy density, ablation rates, plasma condition, and doped internal standard.

Compared to LA-MC-ICP-MS, solution nebulizer MC-ICP-MS has the advantage of high accuracy and precision due to its stable signal and isotopic ratio in the form of solution nebulizer sampling, which makes the target element completely ionized at high temperature (7,500 K) (Human and Scott, 1976). Additionally, a few studies have concentrated on Cu isotope determination of simple chemical matrix without column chromatography (Zhu et al., 2000; Maréchal and Sheppard, 2002; Larson et al., 2003; Mathur et al., 2005, 2009a,b; Balliana et al., 2013; Bao et al., 2020b). The good accuracy and precision have shown that Cu isotope ratios could be determined without column chromatography before mass spectrometry. For example, Zhu et al. (2000) determined the Cu isotopic ratios in native copper, malachite, azurite and chalcopyrite without column chromatography to avoid the chromatographic fractionation. Later, Maréchal and Sheppard (2002) analyzed the malachite without column chromatography by MC-ICPMS. Larson et al. (2003) gained identical Cu isotope ratios with and without column chromatography using SSB method. Mathur et al. (2005) determined the chalcocite, chalcopyrite, bornite without column chromatography. Mathur et al. (2009a,b) measured the chalcocite, chrysocolla, chalcopyrite minerals and cents, and acquired the same results with and without column chromatography using SSB method. However, when SSB method was used to correct mass bias during Cu isotope analysis, instrument drift must be constant, even if absent. Ni internal normalization combined with SSB method was used to correct mass bias and instrument drift (Balliana et al., 2013). Ga has a lower abundance and is less prone to contamination compared with Ni. So, Ga internal normalization combined with SSB method was used to direct measurement of Cu isotope ratios in bronzes (Bao et al., 2020b). Because column chromatography not only consumes too much time, but also requires a large number of experimental materials and reagents (e.g., resin, columns, acid, PFA vails, and Milli-Q water), it is necessary to evaluate whether non-deviated results can be obtained by directly measuring the Cu isotope ratio of Cu-dominated minerals without column chromatography.

In this paper, twelve Cu-dominated minerals (copper plate, native copper, chalcopyrite, bornite, chalcocite, digenite, covellite, tetrahedrite, azurite, malachite, atacamite, cyanotrichite) were analyzed systematically by comparing results between with column chromatography and without column chromatography separately. The matrix effects of nine elements (Co, Fe, Zn, Ge, Al, Mn, Ni, Sb, and Ti) were also evaluated systematically. C-SSBIN mass bias correction model with Ga as an internal standard was selected to acquire high precision Cu isotopic ratios during the process of analysis. Our results indicated that the Cu isotopic ratios with column chromatography are consistent with the results without column chromatography. The non-deviated Cu isotope ratios can be acquired on Cu-dominated minerals without column chromatography.

## Experimental Procedures

### Samples and Reference Materials

Both guaranteed reagent (GR) grade hydrochloric acid (HCl) and nitric acid (HNO_3_) were distilled separately twice in the sub-boiling distillation system (Minnetonka, MN, USA). The ultrapure water with a resistivity of 18.2 MΩ cm^−1^ was acquired from a Milli-Q Element water purification system (Elix-Millipore, Billerica, MA, USA). Two milliliters of Bio-Rad AGMP-1M (100–200 mesh) resin were packed in a polytetrafluoroethylene (Teflon) column with a diameter of 16 mm and a length of 10 cm. All PFA vails were cleaned in GR grade HNO_3_, high-pure HNO_3_, and Milli-Q water prior to use in order.

This study analyzed twelve Cu-dominated minerals, which are copper plate (NWU-Cu-A), native copper, six sulfide minerals including chalcopyrite, bornite, chalcocite, digenite, covellite, and tetrahedrite, and four oxide minerals including azurite, malachite, atacamite, and cyanotrichite. NWU-Cu-A and NWU-Cu-B are the reference materials of Cu used as the in-house Cu standards in the State Key Laboratory of Continental Dynamics (SKLCD), Northwest University, China. The Cu isotope ratios of NWU-Cu-A and NWU-Cu-B are δ^65^Cu = +0.91 ± 0.03‰ and δ^65^Cu = −0.05 ± 0.03‰, respectively, relative to the reference material NIST SRM 976 (Yuan et al., 2017). The NIST SRM 994 Ga was used as an internal standard element to correct the instrumental mass bias, and is certified for ^69^Ga/^71^Ga = 1.50676 ± 0.00039 determined by TIMS (Machlan et al., 1986). Purified concentrated Zn (Alfa Zn, Stock# 13835, Lot# 62-015601C, 1,000 μg g^−1^) and Ni solution (Alfa Ni, Stock# 13839, Lot# 013564M, 1000 μg g^−1^) were purchased from the Johnson Matthey company (London UK). To check the stability of the instrument, three pure Cu standard solutions were used in this work. The three standard solutions include the laboratory Cu standard of the Geochemical Evolution and Metallogeny of Continents Laboratory (GEMOC), Macquarie University, Australia; the GSB Cu standard of the Key Laboratory of Isotopic Geology, Ministry of Land and Resources, China; and the laboratory Cu standard of the Chinese Academy of Geological Sciences (CAGS).

### Sample Digestion Procedure and Ion-Exchange Chromatographic Separation

#### Sample Digestion

All chemical experiments were carried out in the SKLCD. Approximate 2–4 mg of each of the twelve Cu-dominated mineral samples were weighed in the 15 mL pre-cleaned PFA vials. The weighed native copper sample was cleaned firstly with anhydrous alcohol to remove surface contaminants and dissolved in a 3:1 (v/v) mixture of high-pure concentrated HCl–HNO_3_ at 120°C for 2 h in the capped vial. Each of other Cu-dominated minerals was dissolved in 0.4 mL of concentrated HCl–HNO_3_ (3:1, v/v) mixture twice at 80 °C for 12 h in a capped vial to ensure full dissolution. All dissolved samples were evaporated to dryness. Then all dried samples were re-dissolved in concentrated HNO_3_ three times to ensure that the medium is completely converted to HNO_3_ and evaporated to dryness again. Finally, all samples were individually dissolved in 2 mL of 2% HNO_3_ (v/v) for the following experiment.

#### Experimental Design

To evaluate whether non-deviated Cu isotope ratios can be obtained without column chromatography, the Cu-rich solution was divided into two parts for two series of parallel experiments. The first series of experiment took three similar aliquots containing 50 μg of Cu from each Cu-rich sample solution and dealt them with 8 N HCl + 0.001% H_2_O_2_ three times separately. Each of the three aliquots was dissolved in 100 μl of 8 N HCl + 0.001% H_2_O_2_ for the subsequent ion-exchange separation. The Cu solutions collected from column chromatography were dissolved in 2 mL of 2% HNO_3_ (v/v) and analyzed using MC-ICP-MS. The other parallel experiment measured the remaining Cu-rich solutions without column chromatography directly using MC-ICP-MS.

#### Chemical Separation

The column chromatography procedure followed was described in Liu et al. (2014). The resin was alternatively washed with 2 mL of 0.5 N HNO_3_ and 2 mL of MQ H_2_O six times. Twelve milliliters of 8 N HCl + 0.001% H_2_O_2_ was added to the column for conditioning. Then the dissolved sample containing about 50 μg Cu was loaded into the column followed by loading 8 ml of 8 N HCl + 0.001% H_2_O_2_ to elute most matrix elements (e.g., Al, Ca, Ni, Cr, Ti, and Mn). Cu was collected in 22 mL of 8 N HCl + 0.001% H_2_O_2_. Finally, Fe was eluted in the following 20 mL of 2 N HCl. The collected Cu fractions were evaporated to dryness and dissolved in concentrated HNO_3_. Then the dissolved solutions were re-evaporated to dryness. Before Cu isotopic ratio analysis, the solutions were dissolved with 2% HNO_3_ (v/v).

### Mass Spectrometry

The copper isotope ratios were determined by using a double-focusing Nu Plasma II MC-ICP-MS (Nu Instruments, Wrexham, UK) at the SKLCD. This instrument is equipped with sixteen Faraday cups and five full-size discrete dynode multipliers. L6, L5, H3, and H6 Faraday cups were used to collect ^63^Cu, ^65^Cu ^69^Ga, and ^71^Ga, respectively. A “wet” plasma with a wet sampler, skimmer cone, and a quartz nebulizer was used to determine Cu isotope. During the copper isotope analysis, the C-SSBIN with NIST SRM 994 Ga as an internal standard method was selected to correct the instrumental mass bias. The NWU-Cu-B in-house standard solution was served as a bracketing standard. The Cu concentration of samples and bracketing standards were diluted to about 1 μg g^−1^ Cu in 2% HNO_3_ (v/v). The NIST SRM 994 Ga with the concentration of 1 μg g^−1^ was doped in the standards and samples separately. Cu isotope ratios were analyzed in the low-resolution mode (*m*/Δ*m* = 400) and the signal sensitivity of ^63^Cu was about 9 V ppm^−1^. Cu isotope results were expressed as a per mil deviation relative to standard NWU-Cu-B: δ^65^Cu_NWU−Cu−B_ = [(^65^Cu/^63^Cu)_sample_/(^65^Cu/^63^Cu)_NWU−Cu−B_ – 1] × 1000. Finally, all results were converted into relative to standard NIST SRM 976: δ^65^Cu_NISTSRM976_ = δ^65^Cu_NWU−Cu−B_ – 0.05‰ in this study. Major and trace elements were measured by an Agilent 7,900 inductively coupled plasma-mass spectrometry (ICP-MS) at the SKLCD. The analytical uncertainties were better than 10%. Detailed operating parameters of Nu Plasma II are summarized in Table 1.

**Table 1:** Nu Plasma II MC-ICP-MS operating parameters for Cu isotope measurements.

**Instrumental parameters**	**Nu plasma II**
RF power	1,300 W
Cooling gas	13 L min^−1^
Auxiliary gas	0.8 L min^−1^
Nebulizer	39 Psi
^63^Cu sensitivity	9 V ppm^−1^
Background of ^63^Cu	<2 mV
Cones	Ni orifice, wet cone
Resolution mode	Low resolution, *m*/Δ*m* = 400
Mass separation	0.5
Sample uptake	100 μL min^−1^

## Results and Discussion

### Investigation of Matrix Elements and δ^65^Cu Ratios in Cu-Dominated Minerals

The trace elements of the twelve Cu-dominated minerals are reported in Table 2. In general, the Al/Cu, Ti/Cu, Mn/Cu, Co/Cu, Ni/Cu, Sb/Cu, Zn/Cu, and Ga/Cu molar ratios in these minerals were approximate 0, except for Al in cyanotrichite (0.27), Mn in chalcocite (0.027), Sb in chalcocite (0.247), and Zn in chalcocite (0.075) and tetrahedrite (0.054). For Fe element, chalcopyrite has the highest Fe/Cu molar ratio of 0.893. The Fe/Cu molar ratios range from 0.107 to 0.341 in bornite, chalcocite, and tetrahedrite, whereas the Fe/Cu molar ratios were approximate 0 in the other eight Cu-dominated minerals. The Ge/Cu molar ratios were < 0.01 in most minerals, except for chalcopyrite (0.770), bornite (0.188), chalcocite (0.289), and tetrahedrite (0.099). The copper isotope ratios of all Cu-dominated minerals are presented in Table 3, relative to NIST SRM 976. The two standard deviations (2s) were within 0.06‰. All pairs (with and without column chromatography) show similar Cu isotopic ratios with Δδ^65^Cu_without−with_ spanning from −0.04 to +0.02‰ with CSSBIN with Ga internal standard. The Cu isotope ratios of a copper plate (NWU-Cu-A) is δ^65^Cu = 0.91 ± 0.04‰ (2s, *n* = 52), identical with the reported values of 0.91 ± 0.03‰ (Yuan et al., 2017).

**Table 2:** Trace elements molar ratios of Cu-dominated minerals.

**Minerals**	**Al/Cu**	**Ti/Cu**	**Mn/Cu**	**Fe/Cu**	**Co/Cu**	**Ni/Cu**	**Ge/Cu**	**Sb/Cu**	**Zn/Cu**	**Ga/Cu**
Chalcopyrite (CuFeS_2_)	0.000	0.000	0.002	0.893	0.000	0.000	0.770	0.003	0.006	0.000
Bornite (Cu_5_FeS_4_)	0.001	0.000	0.000	0.204	0.000	0.000	0.188	0.000	0.002	0.000
Chalcocite (Cu_2_S)	0.001	0.000	0.027	0.341	0.000	0.000	0.289	0.247	0.075	0.000
Digenite (4Cu_2_S·CuS)	0.000	0.000	0.000	0.011	0.000	0.000	0.009	0.000	0.000	0.000
Covellite (CuS)	0.000	0.000	0.000	0.002	0.000	0.000	0.003	0.000	0.000	0.000
Tetrahedrite (Cu_12_Sb_4_S_13_)	0.003	0.000	0.001	0.107	0.000	0.000	0.099	0.016	0.054	0.000
Azurite [Cu_3_(CO_3_)_2_(OH)_2_]	0.002	0.000	0.000	0.003	0.000	0.000	0.004	0.000	0.002	0.000
Malachite [Cu_2_(OH)_2_CO_3_]	0.003	0.000	0.002	0.002	0.000	0.000	0.005	0.000	0.002	0.000
Atacamite (Cu_2_(OH)_3_Cl)	0.001	0.000	0.000	0.004	0.000	0.000	0.007	0.000	0.000	0.000
Cyanotrichite [Cu_4_Al_2_(SO_4_)(OH)_12_·2H_2_O]	0.270	0.000	0.000	0.000	0.000	0.000	0.003	0.002	0.001	0.000
Copper palte (NWU-Cu-A) (Cu)	0.000	0.000	0.000	0.000	0.000	0.000	0.000	0.000	0.000	0.000
Native copper (Cu)	0.003	0.000	0.000	0.004	0.000	0.000	0.003	0.000	0.000	0.000

**Table 3:** Copper isotope ratios of Cu-dominated minerals with/without column chromatography.

**Minerals**	**δ^65^Cu**_**with**_	**2s**	**C-SSBIN with Ga internal standard**			**C-SSBIN with Zn internal standard**			**C-SSBIN with Ni internal standard**		
			**δ^65^Cu**_**without**_	**2s**	***Δδ*^65^Cu**_**without-with**_	**δ^65^Cu**_**without**_	**2s**	***Δδ*^65^Cu**_**without-with**_	**δ^65^Cu**_**without**_	**2s**	***Δδ*^65^Cu**_**without-with**_
Chalcopyrite	0.04	0.02	0.04	0.04	0.01	0.04	0.05	0.00	0.08	0.04	0.04
Bornite	−1.23	0.01	−1.27	0.02	−0.04	−1.18	0.04	0.05	−1.24	0.04	−0.02
Chalcocite	−0.06	0.02	−0.07	0.03	−0.01	−0.18	0.03	−0.12	−0.04	0.05	0.02
Digenite	0.09	0.03	0.09	0.02	−0.01	0.10	0.05	0.01	0.08	0.03	−0.01
Covellite	0.29	0.02	0.28	0.04	−0.01	0.27	0.04	−0.02	0.31	0.02	0.02
Tetrahedrite	−0.17	0.05	−0.22	0.03	−0.04	−0.05	0.00	0.12	−0.18	0.05	−0.01
Azurite	0.99	0.01	0.99	0.01	0.00	1.02	0.03	0.02	1.03	0.05	0.04
Malachite	0.64	0.00	0.66	0.02	0.02	0.67	0.02	0.02	0.68	0.04	0.03
Atacamite	0.72	0.01	0.69	0.04	−0.02	0.70	0.06	−0.01	0.73	0.04	0.02
Cyanotrichite	1.65	0.05	1.61	0.04	−0.04	1.65	0.03	−0.01	1.66	0.06	0.01
Copper plate	0.91	0.00	0.90	0.03	−0.01	0.92	0.03	0.01	0.90	0.03	−0.01
Native copper	0.45	0.00	0.46	0.03	0.02	0.48	0.02	0.04	0.47	0.05	0.02
Chalcopyrite (tc17–25)	−0.05	0.03	−0.08	0.03	−0.03						

*δ^65^Cu_without_ and δ^65^Cu_with_ denote Cu isotope ratios of minerals that were processed with column and without column chromatography respectively. 2s is 2 standard deviations. Δδ^65^Cu_without−with_ = δ^65^Cu_without_ – δ^65^Cu_with_*.

### Effects of Acidity and Internal Standard Concentration Mismatch

**Figure 1:**
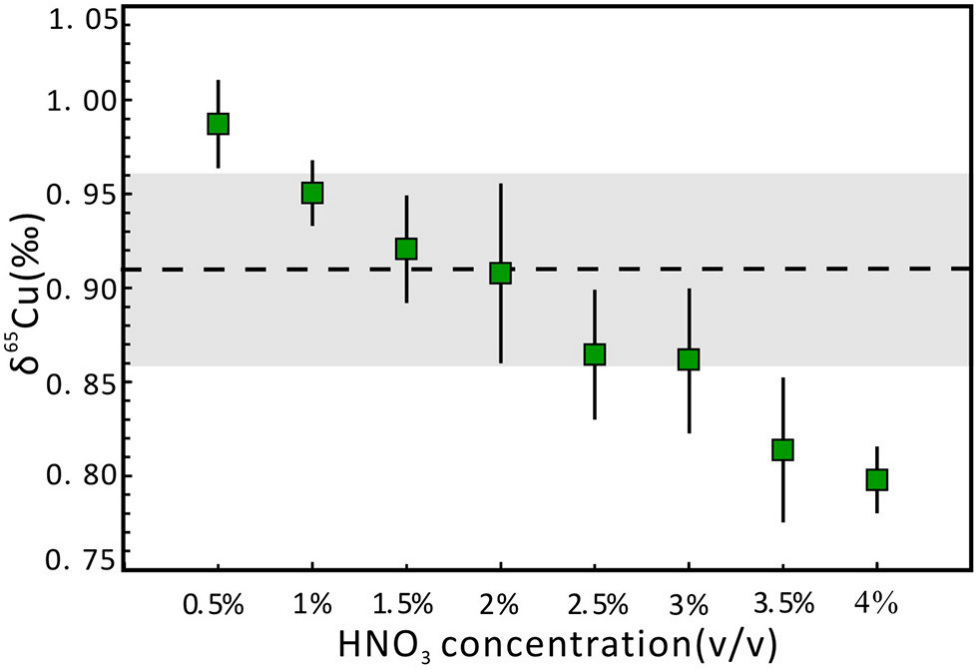
Effect of different acidities of NWU-Cu-A solution on δ^65^Cu. Precisions are expressed as 2 standard deviations (2s), which were calculated on the basis of three or four replicate measurements. The dotted line indicates the reference value obtained by Yuan et al. (2017). The gray area represents the 0.05‰ uncertainty range.

The metal stable isotopic deviation can be affected by acidity mismatch in the SSB method, even using standard-sample bracketing with Zn or Ni as the internal standard (Liu et al., 2014; Yuan et al., 2017). In this study, we used 1 μg g^−1^ NWU-Cu-B solution diluted with 2% (v/v) HNO_3_ as the bracketing standard for Cu isotope analysis. A series of 1 μg g^−1^ NWU-Cu-A solutions with 0.5–4.0% (v/v) HNO_3_ were used to evaluate the effect of acidity. The SSB method combined with the use of Ga as the internal standard has been employed to correct instrumental mass bias during Cu isotope analysis. The results display that the inconsistency of acidity between the standard and the sample solutions has an important influence on the Cu isotopic ratios in the wet plasma mode (Figure 1). There is a strong negative correlation between the δ^65^Cu values and the acidity. When the acidity was from 1.0 to 3.0% (v/v), the δ^65^Cu values were in agreement with the reference value within the 0.05‰ uncertainty intervals. When the HNO_3_ acidity was between 1.5 and 2.0% (v/v), the results were consistent with a reference value within the 0.01‰ uncertainty. Moreover, with the acidities of 3.5 and 4.0‰, the δ^65^Cu values were 0.81 and 0.80‰, respectively, lighter than the δ^65^Cu value of 0.91‰ with the acidity of 2% (v/v). This phenomenon is likely caused by the polyatomic interferences when using HNO_3_ as a medium during the testing process with MC-ICP-MS (Zhu et al., 2015). Therefore, to avoid the effect of acidity, the sample and standard solutions should be diluted with the same 2% (v/v) HNO_3_.

**Figure 2:**
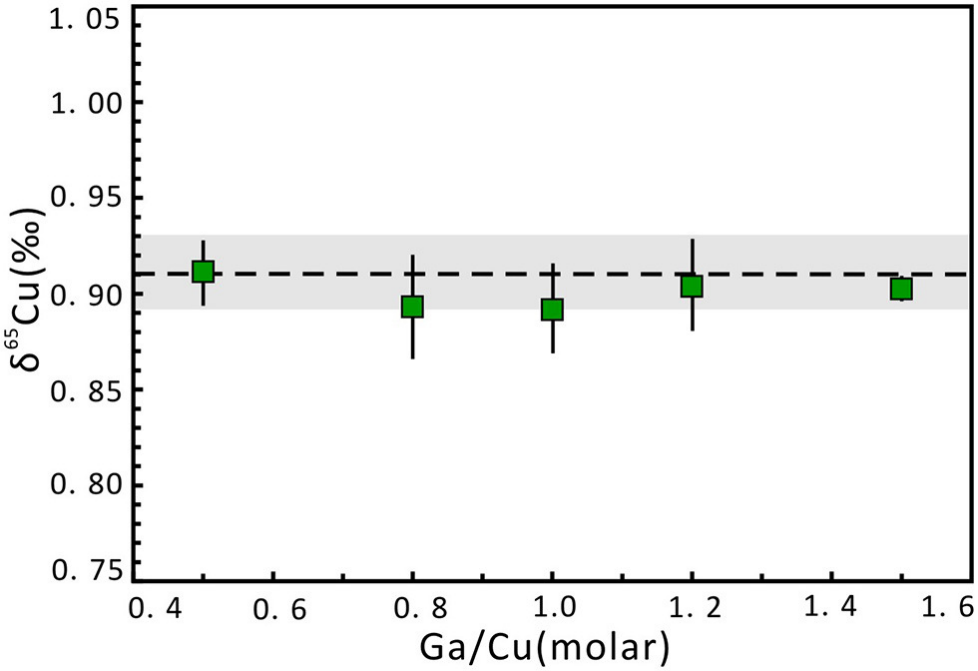
Cu isotope ratio variation of NWU-Cu-A solution with changing Ga concentration relative to Cu (1 μg g^−1^) in the sample and bracketed standard (NWU-Cu-B). Errors are presented at the 2s level, which was obtained by measuring three times. The dotted line indicates the δ^65^Cu reference value acquired by Yuan et al. (2017). The gray region expresses the 0.02‰ uncertainty range.

The effect of Ga concentration on Cu isotope ratios was evaluated by doping different proportional concentrations of Ga relative to Cu in the NWU-Cu-B and NWU-Cu-A, while the Cu concentrations of sample and standard solution were 1 μg g^−1^. As shown in Figure 2, the concentration of Ga does not affect the accuracy of Cu isotopic measurements. For Ga/Cu ratios varied from 0.5 to 4.0, the δ^65^Cu values agreed well with the reference value within 0.02‰ uncertainty. Therefore, the effect of different Ga concentrations on Cu isotope ratios is negligible. To avoid the mass discrimination induced by doping different Ga concentrations, Ga concentration in samples and bracketing standard was set to 1 μg g^−1^, identical to the Cu concentration.

### Effects of Matrix Elements

**Figure 3:**
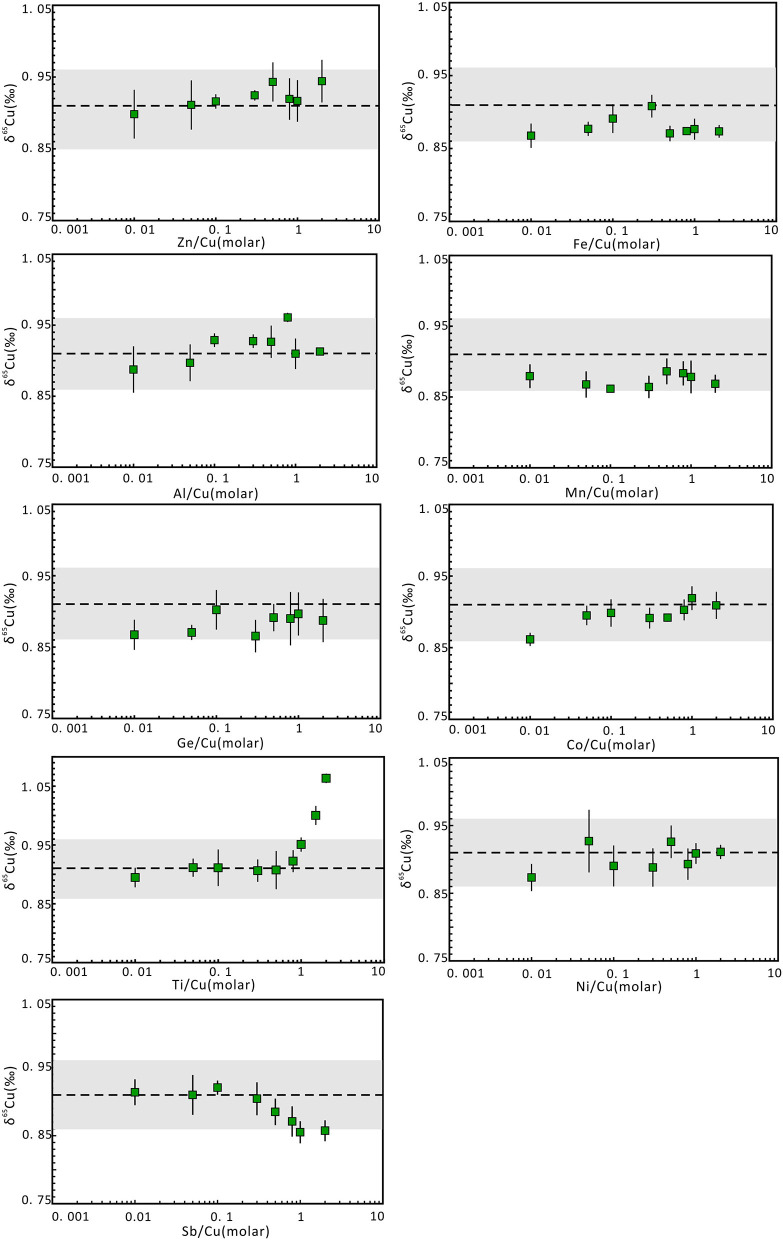
Cu isotope ratio variation of NWU-Cu-A solutions with doping different proportions of matrix elements relative to Cu. The Cu concentration of samples and bracketed standards (NWU-Cu-B) is the same (1 μg g^−1^). The errors were obtained by three or four replicate measurements. The dotted line represents the δ^65^Cu reference value given by Yuan et al. (2017). The gray region represented the 0.05‰ uncertainty range.

The existence of matrix elements can lead to matrix effects and affect the determination of ^65^Cu/^63^Cu ratios. To evaluate the effects of undesired matrix elements in Cu-dominated minerals, matrix elements that may be present in the Cu solution without column chromatography were individually doped to pure Cu solution of NWU-Cu-A with different matrix elements/Cu molar ratios, such as 0.01, 0.05, 0.1, 0.3, 0.5, 0.8, 1, and 2. The results of these analyses were plotted in Figure 3. For most matrix elements (such as Co, Fe, Zn, Ge, Al, Mn, Ni, Sb) with matrix elements/Cu molar ratios up to 2, the δ^65^Cu values were consistent with the reference value within the 0.05‰ uncertainty, and the influence of these elements can be ignored during the measurement. Furthermore, Hou et al. (2016) showed that the influence of Fe on Cu isotope ratios can be neglected with the Fe/Cu molar ratios up to 20 when using the Neptune MC-ICPMS with C-SSBIN method. In the twelve Cu-dominated minerals solutions, the measured Fe/Cu, Ge/Cu, Al/Cu, Sb/Cu, Zn/Cu, Mn/Cu ratios were <0.9, 0.8, 0.3, 0.3, 0.08, 0.03, respectively, and the Ni/Cu and Co/Cu ratios were approximately 0. Therefore, the effect of these matrix elements on the final Cu isotopic ratios can be neglected during the measurement without column chromatography.

The matrix effect of Ti on Cu isotope ratios is also shown in Figure 3. It is obvious that when the Ti/Cu ratio is <1, accurate δ^65^Cu values can be obtained within 0.04‰ uncertainty. With Ti/Cu ratios of 1.5 and 2.0, the δ^65^Cu values were 1.0 and 1.06‰, respectively, heavier than the reference value of NWU-Cu-A (0.91‰). The mass discrimination of Cu isotope was caused by the polyatomic inferences of ^47^Ti^16^O^+^ and ^46^Ti^16^O^1^H^+^ on ^63^Cu as well as ^49^Ti^16^O^+^ and ^48^Ti^16^O^1^H^+^ on ^65^Cu (Mason et al., 2004). Moreover, ^65^Cu was more susceptible to Ti polyatomic inferences compared to ^63^Cu. Nonetheless, the Ti polyatomic inferences are negligible for measurement without chromatography, because the Ti concentration was low in Cu-dominated minerals solutions without column chromatography and the determined Ti/Cu ratios were approximate 0.

### Comparison of the C-SSBIN With Ga as an Internal Standard With Other Methods

As mentioned above, different mass bias correction models have been reported, such as the SSB method and the internal normalization using Ni, Zn, or Ga combined with the SSB method. The key to using the C-SSBIN method during the Cu isotope analysis is to completely remove the internal standard element from the final solution. The residual of the internal standard element interferes with the adopted inter-element, which may cause varied isotopic ratios of the internal standard element and further lead to an overcorrection for the determined δ^65^Cu values. Unfortunately, internal standard elements (e.g., Zn and Ni) may exist in Cu-dominated mineral solutions without column chromatography. To obtain accurate Cu isotopic ratios, it is essential to evaluate the Cu isotope ratios of Cu-dominated minerals using different correction models. The results of all Cu-dominated minerals corrected by the C-SSBIN method with Ga, Zn, and Ni as respective internal standard are shown in Table 3.

During Cu isotope analysis using the SSB method, most of the single measurement uncertainties of δ^65^Cu ranged from 0.04 to 0.10‰. When Ga internal normalization combined with the SSB method was used to correct the mass bias and instrument drift, the single measurement uncertainties were mostly better than 0.04‰. The mean δ^65^Cu values for NWU-Cu-A and tc17-25 during different analytical sessions using direct the SSB method were 0.91 ± 0.08‰ (2s, *n* = 50) and −0.08 ± 0.07‰ (2s, *n* = 38), respectively. These results indicate that both mass bias correction methods could generate accurate Cu isotopic ratios, but the precision was improved by two times by using the C-SSBIN with Ga as an internal standard. When Zn was used as an internal standard, ten Cu-dominated minerals with low abundances of Zn displayed little drift in δ^65^Cu values, with Δδ^65^Cu_without−with_ ranging from −0.02 to +0.05‰. However, a larger deviation in δ^65^Cu values was observed in chalcocite and tetrahedrite samples due to the residual Zn in the final solutions without column chromatography. The Δδ^65^Cu_without−with_ values were −0.12 and +0.12‰, with Zn/Cu ratios of 0.075 and 0.054, respectively. The Δδ^65^Cu_without−with_ ranged from −0.02 to +0.04‰ when the C-SSBIN with Ni as an internal standard was used. Due to the low abundances of Ni in these Cu-dominated minerals, it is reasonable that the accuracy of δ^65^Cu is similar to that obtained using the C-SSBIN with Ga as an internal standard. Considering the possible existence of Ni in the actual samples or the background produced by the Ni cone, Ga is more suitable as an internal standard element. As a result, the C-SSBIN with Ga as an internal standard method was selected to improve the accuracy and precision of determination of Cu isotope ratios in Cu-dominated minerals without column chromatography.

### Cu Isotopic Ratio Measurement Without Column Chemistry on Cu-Dominated Minerals

To obtain accurate and precise isotope ratios of natural samples, the matrix elements should be reduced or eliminated prior to mass spectrometry. In fact, this is may be true for most isotope systems, otherwise, matrix effects and isobaric interferences will cause mass discrimination, such as for Mg (An et al., 2014; Bao et al., 2019), Ca (Feng et al., 2018; Bao et al., 2020a), Zr (Feng et al., 2020), Ba (Nan et al., 2015), and V (Wu et al., 2016). Nevertheless, matrix effects can be significantly reduced during MC-ICP-MS analysis for two considerations: (a) high Cu concentration in Cu-dominated minerals and (b) using a C-SSBIN with Ga as internal standard technology to correct the instrumental mass bias. For some stable systems in samples with simple matrix, if isobaric interferences and matrix effect can be well-constrained, accurate and precise isotopic data would be obtained without chemical chromatography. For example, Balliana et al. (2013) suggested that direct isotopic analysis of Cu on ancient bronzes with MC-ICP-MS without chemical separation seems to be feasible, and shows no evidence of spectral interference or matrix effect on the degree of mass bias. Bao et al. (2020b) suggest that direct measurement of Cu isotope ratios without column chromatography is possible by using Ga as internal normalization.

**Figure 4:**
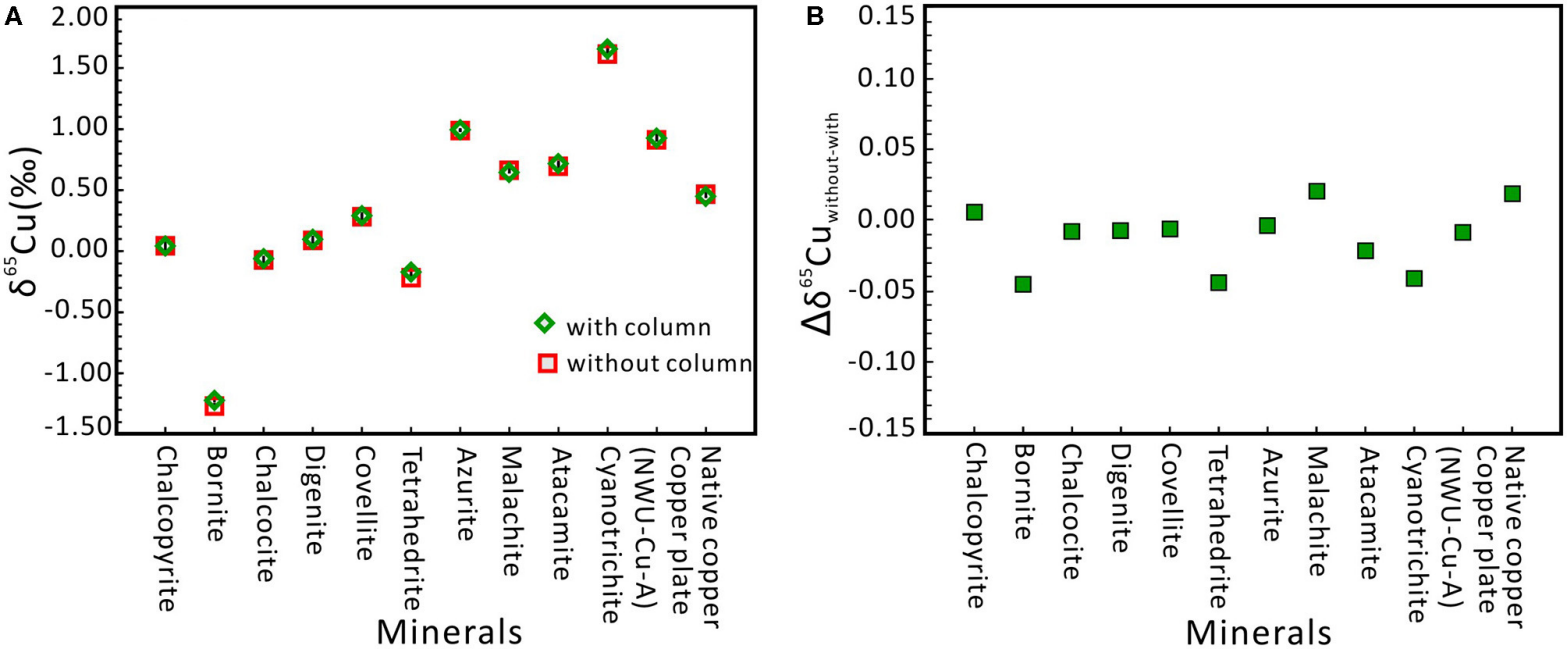
δ^65^Cu values for twelve Cu-dominated minerals with and without column chromatography, respectively. **(A)** Cu isotope ratios of all samples without column chromatography agree well with the ratios with column chromatography. Errors are presented at the 2s level and obtained through three or four times of measurement. **(B)** Comparison of Cu isotope ratios with column and without column chromatography. Δδ^65^Cu_without−with_ of twelve Cu-dominated minerals ranged from −0.04 to +0.02‰.

During the MC-ICP-MS determination of Cu isotope, matrix elements (such as Al, Ti, Mn, Fe, Co, Ni, Ge, Sb, and Zn) have limited effects on Cu isotope ratios (see section Effects of Matrix Elements), and thus we propose that Cu-dominated materials can be directly measured by MC-ICP-MS without column chromatography using a C-SSBIN with Ga as internal standard technology. As shown in Figure 4, the difference between with and the without chemical separation is very small, with the Δδ^65^Cu_without−with_ ranging from −0.04 to +0.02‰. The Δδ^65^Cu_without−with_ values of the remaining nine minerals samples are from −0.02 to 0.02‰. This proposed method can guarantee the accuracy of the measured Cu isotopic ratios for Cu-dominated minerals. Moreover, within the analytical error, if the without-chemical-chromatography method can meet the need for research, this methodology can not only simplify the sample handling procedure and save time but also reduce reagent consumption and expenses. Therefore, the Cu-dominated minerals (including copper plate, native copper, chalcopyrite, bornite, chalcocite, digenite, covellite, tetrahedrite, azurite, malachite, atacamite, and cyanotrichite) can be determined directly by using MC-ICP-MS without column chromatography.

### Quality Control of the Cu Isotope Ratios of Samples

**Figure 5:**
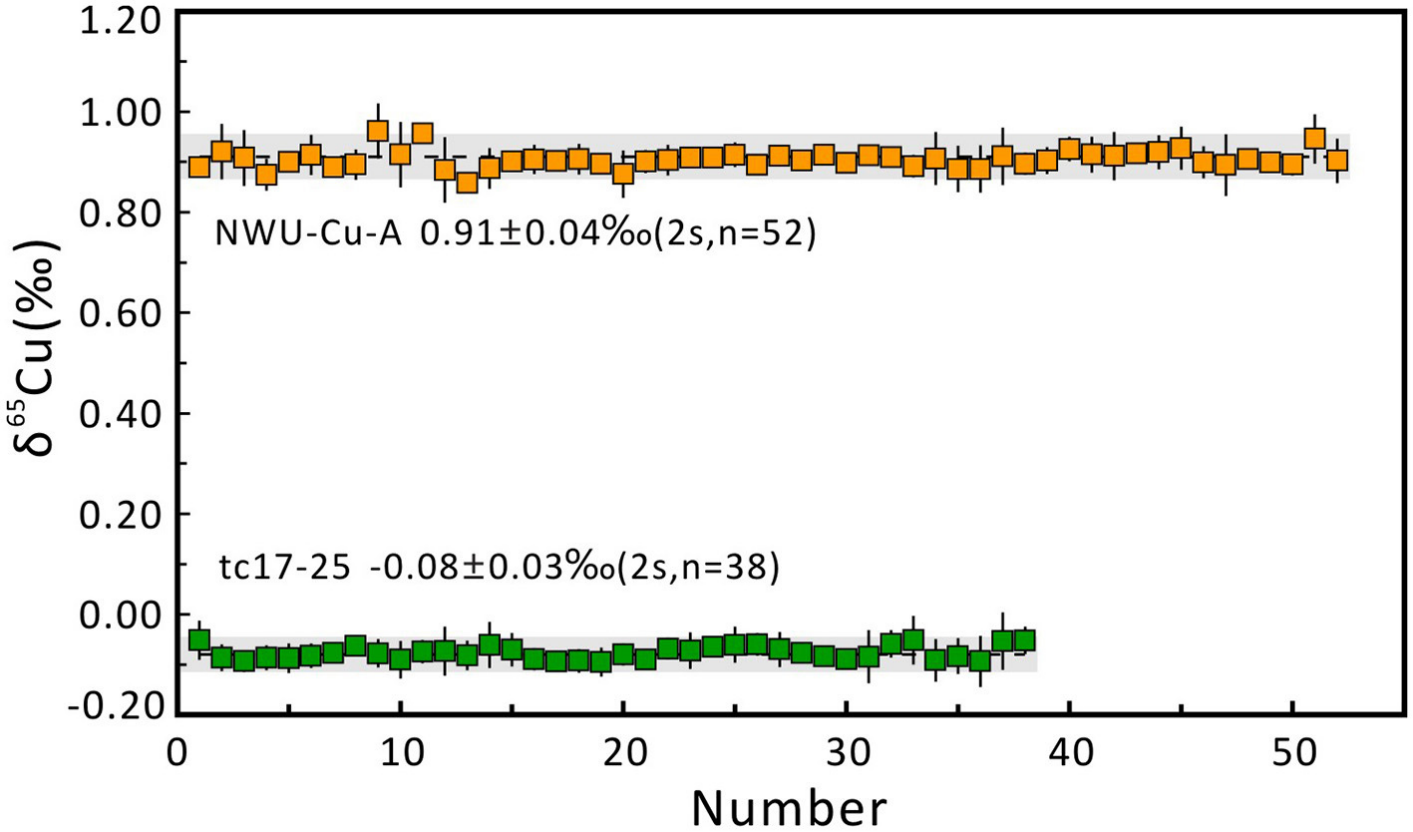
Long-term reproducibility of δ^65^Cu measurement of chalcopyrite (tc17-25) and NWU-Cu-A relative to NWU-Cu-B. The precision was better than 0.03‰ (2s) for tc17-25 over 4 months and 0.04‰ (2s) for NWU-Cu-A over 5 months.

Five aliquots of NWU-Cu-A standard solution doped with matrix elements (e.g., Fe, Zn, Al, Ti, Co, Ni, Ge, Sb, Mn) were determined to check the purification process and mass spectrometry to evaluate the data quality. Besides, some pure Cu standard solutions containing GEMOC Cu, GSB Cu, and CAGS Cu were repeatedly measured in this study. The results are shown in Table 4. Relative to NIST SRM 976, the NWU-Cu-A solution doped with matrix elements yielded a mean value of 0.91 ± 0.05‰ (2s, *n* = 5), identical with the reported value of the pure solution (Yuan et al., 2017). The δ^65^Cu ratios of GEMOC Cu, GSB-Cu, and CAGS Cu standard solutions are identical with the previously reported values within 2s uncertainties (Hou et al., 2008; Liu et al., 2014; Yuan et al., 2017; Li et al., 2019). In this study, the long-term analytical precision of Cu isotopic ratios was evaluated by repeatedly analyzing pure NWU-Cu-A standard solution and chalcopyrite (tc17-25) solution without column chromatography using MC-ICP-MS. The Cu isotope ratios of NWU-Cu-A and tc17-25 without column chromatography shown in Figure 5 show the variation range of Cu isotope ratios is small within 5 months and the long-term precision of the δ^65^Cu is ±0.04‰. Relative to NIST SRM 976, the NWU-Cu-A solutions over 5 months gave a mean δ^65^Cu of 0.91 ± 0.04‰ (2s, *n* = 52), also identical within 2s uncertainty with the values obtained by Yuan et al. (2017). For tc17-25 solution without column chromatography, an average value of δ^65^Cu = −0.08 ± 0.03‰ (2s, *n* = 38) was obtained over 4 months. For Cu-dominated samples directly determined during this study, the long-term reproducibility was better than ±0.04‰, which is comparable to other studies (Maréchal et al., 1999; Chapman et al., 2006; Hou et al., 2008; Larner et al., 2011; Liu et al., 2014). Thus, the purification procedure and the MC-ICP-MS measurements proposesd were robust and replicable with good accuracy and precision in this study.

**Table 4:** Copper isotope ratios for NWU-Cu-A, CAGS Cu, GSB Cu, GEMOC Cu standard solutions.

**Samples**	**δ^65^Cu (‰)**	**2s**	***n***	**Comments/references**
NWU-Cu-A	0.91	0.05	5	This study
	0.91	0.03	42	Yuan et al., 2017
CAGS Cu	0.53	0.03	9	This study
	0.55	0.05	9	Hou et al., 2008
	0.54	0.07	17	Yuan et al., 2017
	0.53	0.08	27	
	0.57	0.06	18	Li et al., 2019
GSB Cu	0.45	0.05	13	This study
	0.44	0.04	32	Liu et al., 2014
	0.47	0.06	12	Yuan et al., 2017
	0.44	0.07	24	
GEMOC Cu	−0.02	0.01	2	This study
	−0.04	0.07	10	Hou et al., 2008
	−0.01	0.07	46	Yuan et al., 2017
	−0.02	0.05	54	

*n is the number of repeat analysis drafted the manuscript*.

The data quality of Cu isotope ratio results can also be affected by the total procedural Cu blank and recovery. The total procedural Cu blank during sample dissolution and chromatographic procedures was minimized by using Milli-Q H_2_O and double distilled acids. The total procedural Cu blank was <3 ng, which is negligible compared with the 50 μg Cu loaded into the column. The Cu recovery ranged from 98.8 to 99.7% during the total procedure for the twelve samples. Such a good recovery was also verified by accurately and precisely determining δ^65^Cu ratios of NWU-Cu-A solution doped with matrix elements.

## Conclusions

In this study, twelve Cu-dominated minerals (copper plate, native copper, chalcopyrite, bornite, chalcocite, digenite, covellite, tetrahedrite, azurite, malachite, atacamite, and cyanotrichite) were analyzed by Nu II Plasma MC-ICP-MS with and without column chromatography. For these samples, we have found the Cu isotopic ratios without column chromatography are in good agreement with Cu isotopic ratios with column chromatography, with the Δδ^65^Cu_without−with_ values ranging from−0.04 to +0.02‰.

Because the acidity mismatch in the media between the sample and standard solution considerably affects the isotopic ratio, the medium acidity of the sample must be the same as that of the standard solution. However, differences in the internal standard concentration barely influence the measurements of the Cu isotopic composition. The influence of matrix elements on the final Cu isotopic ratios can be neglected by using a C-SSBIN with Ga as the internal standard technology. Note the Ti/Cu molar ratio should be <1.0 to guarantee the accuracy of Cu isotope analysis. The Δδ^65^Cu values of the twelve Cu-dominated minerals show insignificant differences between solutions with and without column chromatography. The Δδ^65^Cu_without−with_ values spanning from −0.04 to +0.02‰ in these samples have validated the reliability of the proposed method. Repeat analysis of the tc17-25 solution without column chromatography, the external reproducibility of δ^65^Cu is better than 0.03‰ (2s). Therefore, this proposed method has a significant advantage for the economical and efficient determination of Cu isotopic ratios of Cu-dominated minerals.

## Data Availability Statement

The raw data supporting the conclusions of this article will be made available by the authors, without undue reservation.

## Author Contributions

HY and ZB designed the experiment. YZ and ZB performed the experiment and drafted the manuscript. NL, KC, and CZ performed manuscript review. All the authors have read and approved the content of the manuscript.

## Conflict of Interest

The authors declare that the research was conducted in the absence of any commercial or financial relationships that could be construed as a potential conflict of interest.
